# 

**Published:** 1840-10

**Authors:** 


### BOOKS RECEIVED FOR REVIEW.

### ENGLISH.

1. Statistical Reports of the Sickness, Mortality, and Invaliding among the Troops in Western Africa, St. Helena, the Cape of Good Hope, and the Mauritius; prepared from the records in the army medical department and war-office returns. By Major Tulloch.—London, 1840. Fol.

2. The Principles of Botany; structural, functional, and systematic. For the use of students in medicine. By W. H. Willshire, M.D.—London, 1840. Sm. 8vo, pp. 232. 6s.

3. Transactions of the Medical and Physical Society of Bombay. Vol. II.—Bombay, 1839. 8vo, pp. 269.

4. First Principles of Surgery, being an Outline of Inflammation and its effects. By George T. Morgan, A.M., formerly Lecturer on Surgery in Aberdeen.—London, 1840. 8vo, 18s. Part III., 7s. 6d.

5. Practical Observations on Distortions of the Spine, Chest, and Limbs; together with remarks on paralytic and other diseases connected with impaired or defective motion. By W. T. Ward, F.L.S., M.R.C.S. —London, 1840. 8vo, pp. 202. 7s.

6. Anatomical Sketches and Diagrams. By Messrs. Wormald and M'Whinnie. Part III. 4s.

7. On Diseases of the Bladder and Prostate Gland. With Plates. By William Coulson. Second Edition, greatly enlarged.—London, 1840. 8vo, pp. 258. 7s.

8. Guy's Hospital Reports. No. X. April, 1840. 8vo, pp. 184. 6s.

9. Dr. Ramsbotham's Atlas of Obstetric Plates. Nos. IV. V. VI. VII. VIII. 1840. 1s. 6d.

10. Odontography; or a Treatise on the Comparative Anatomy of the Teeth; their physiological relations, mode of development, and microscopic structure in the vertebrate animals, illustrated by upwards of 150 Plates. By Richard Owen, F.R.S., Hunterian Professor to the Royal College of Surgeons, London. Part I.—London, 1840. Royal 8vo, pp. 112. Fifty Plates. £1 11s. 6d.

11. The Retrospective Address in Surgery, from July 1836 to July 1839, delivered before the Meeting of the Provincial Medical Association at Liverpool on the 24th July. By J. H. James, Esq., Surgeon to the Devon and Exeter Hospital.—Worcester, 1840. 8vo, pp. 92.

12. Müller's Elements of Physiology. Translated from the German, with Notes. By W. Baly, M.D. Second Edition. Vol. I. 8vo, pp. 848. 20s.

13. The Library of Medicine. Arranged and edited by Alexander Tweedie, M.D., F.R.S., &c. Practical Medicine. Vols. I. II. III. IV.—London, 1840. 8vo, pp. 440, 353, 385, 361. 10s. 6d. each.

14. A Treatise on Amaurosis and Amaurotic Affections. By Edward Octavius Hocken.—Lond. 1840. 8vo, pp. 359. 10s. 6d.

15. First Lines of Education: a Course of Four Lectures, delivered to the Literary and Scientific Institution, Worcester. By E. A. Turley, Surgeon.—Worcester, 1839. 8vo, pp. 84.

16. Observations on Longevity and on Public Health, read at the Worcestershire Natural History Society, Jan. 22, 1840. By Ch. Hastings, M.D., &c.—Worcester, 1840. 8vo, pp. 27. 1s.

17. A Practical Work on the Diseases of the Eye, and their treatment, medically, topically, and by operation. By Frederick Tyrrell, Senior Surgeon of the London Ophthalmic Hospital, Surgeon to St. Thomas's Hospital, &c. Two Volumes, 8vo, pp. 533, 545. With Plates. £1 16s.

18. Commentaries on Diseases of the Skin; illustrated by coloured plates, drawn by Joseph Perry. By A. T. Thomson, M.D., &c. Fasc. I. II. III. 1840. 10s. 6d.

19. Parochial Medical Relief, considered in a Letter to the Poor Law Commissioners, developing an entirely new system of medical remuneration. By E. T. Meredith, Surgeon.—London, 1840. 8vo, pp. 35. 1s. 6d.

20. On the Nature and Structural Characteristics of Cancer, and of those morbid growths which may be confounded with it. By J. Müller, M.D., Professor, &c.—Berlin. Translated from the German, with Notes, by Charles West, M.D., &c. With numerous engravings. — London, 1840. Part I. 8vo, pp. 182. 7s. 6d.

21. The Twenty-first Report of the Director (C.C. Corsellis, M.D.) of the West Riding of York Pauper Lunatic Asylum. —Wakefield, 1840. 8vo, pp. 16.

22. A Letter to Sir B. C. Brodie, containing a critical enquiry into his "Lectures illustrative of certain local Nervous Affections." By William Goodlad, Surgeon.—London, 1840. 8vo, pp. 154.

23. Observations on the Diseases incident to Pregnancy and Childbed. By F. Churchill, M.D., &c.—Dublin, 1840. 8vo, pp. 463. 12s.

24. A Treatise on the Physiological and Moral Management of Infancy. By Andrew Combe, M.D., &c.—Edinburgh, 1840. 12mo, pp. 375. 6s.

25. Practical Observations on Abortion. By J. F. Streeter, Surgeon. With Plates and Woodcuts.—Lon., 1840. 8vo, pp. 70. 5s.

26. The Transactions of the Provincial Medical and Surgical Association. Vol. VIII. With 35 Coloured Plates.—London, 1840. 8vo, pp. 434. £1 11s. 6d.

27. Illustrations of Cutaneous Disease. Parts XVI. XVII. XVIII. By R. Willis, M.D.—London, 1840. 5s.

28. A practical Essay on the disease generally known under the denomination of Delirium Tremens, written principally with a view to elucidate its divisions into distinct stages, and hence to simplify its method of cure. By A. Blake, M.D. &c. Second Edition, revised and much enlarged. London, 1840. 8vo. pp. 112. 5s.

29. An account of the Yellow Fever which appeared in the city of Galveston, Republic of Texas, in 1839, with cases and dissection. By A. Smith, M.D., A.M., Ex-Surgeon General of the Texian Army. Hounston, 1839. 8vo, pp. 78.

30. The Cyclopædia of Surgery. Edited by W. B. Costello, M.D. Part VI. Burns—Cancer.—London. June, 1840. 5s.

31. The Cyclopædia of Anatomy and Physiology. Edited by R. B. Todd, M.D. F.R.S. Parts XIX. XX. Instinct—Liver.—London. July, 1840. 5s.

32. Cursory Notes on the Morbid Eye. By Robert Hull, Physician to the Norfolk Hospital.—Lon., 1840. 8vo, pp. 249. 8s.

33. A Supplement to his Statements to Mr. Warburton in the Parliamentary Committee. By W. Stokes, M.D.—Dublin, 1840. 8vo, pp. 12.

34. A reply to the Pamphlet, entitled "Proposed Alterations in the Scottish Poor Law." By W. P. Alison, M.D. Edinburgh, 1840. 8vo, pp. 75.

35. The Philosophy of Instinct and Reason. By J. S. Bushnan, M.D. F.L.S. —Edinburgh, 1837. 8vo, pp. 316. With Eight Plates. 5s.

36. On the best method of obtaining the more powerful Vegetable Preparations for Medical Use. By E. Bentley. 8vo, pp. 10.

37. Twentieth Annual Report of the Directors of the Dundee Lunatic Asylum. —Dundee, 1840. 8vo, pp. 39.

38. Report of the Montrose Lunatic Asylum, Infirmary, and Dispensary for 1840.—Montrose, 1840. 8vo, pp. 54.

39. An Address upon Laying the Foundation Stone of the Queen's Hospital, Birmingham. June 18, 1840: with Notes and an Appendix. By Vaughan Thomas, B.D.—Oxford, 1840. 8vo, pp. 78.

40. A Letter to James Law, A.M., on the importance of establishing, in connexion with the Birmingham School of Medicine, a Clinical Hospital. By W. S. Cox, F.R.S. 8vo, pp. 28.

41. The Aortic System, anatomically and physiologically considered, &c., being the Warneford Prize Essay for 1840. By E. Smith, Student of the Birmingham School of Medicine.—Birmingham, 1840. 8vo, pp. 91.

42. Trifles from my Portfolio, or recollections of scenes and small adventures during twenty-nine years' military service. By a Staff Surgeon.—Quebec, 1839. Two vols. 8vo, pp. 251-52. 20s.

43. A Probationary Essay on Tobacco. By H. W. Cleland, M.D. Lecturer on Medical Jurisprudence.—Glasgow, 1840. 4to. pp. 65.

44. The Baths of Nassau, Baden, and the adjacent districts, considered with reference to their remedial efficacy in Chronic Disease. By Edwin Lee, esq. M.R.C.S.—Frankfort and Wisbaden, 1840. 8vo, pp. 172. 5s. 6d.

45. Acute Hydrocephalus, or Water in the Head; an inflammatory disease, and curable equally and by the same means with other diseases of inflammation. By D.D. Davis, M.D., Professor of Medicine in University College.—London, 1840. 8vo, pp. 309. 9s. 6d.

46. The Annual Address to Candidates for Degrees and Licences in the Medical Institution of Yale College, Jan. 1, 1840. By D. T. Brainard, M.D.—New Haven, 1840. 8vo, pp. 16.

47. Medical and Physiological Commentaries. By Martyn Paine, M.D. A.M. Two thick vols.—New York, 1840. 8vo, 36s.

48. A Letter on the Efficacy of Mineral Waters in the Treatment of Chronic Disorders. By J. Pickford, Surgeon to the Brighton Eye Infirmary.—Brighton, 1840. 8vo, pp. 46. 1s.

49. The Retrospect of Practical Medicine and Surgery, for the year 1840. No. I. Jan.—July. By W. Braithwaite, Surgeon. —London, 1840. Sm. 8vo, pp. 200. 4s. 6d.

50. Surgical, Operative, and Mechanical Dentistry. By L. C. De Londe. With 4 Plates.—Lon. 1840. 8vo, pp. 185. 12s. 6d.

51. Lectures on the Morbid Anatomy of the Serous and Mucous Membranes. Vol. II. Part I. On the Mucous Membranes.—By T. Hodgkin, M.D.—London, 1840. 8vo, pp. 541. 12s.

52. A Treatise on the Eye, containing discoveries of the causes of near and far sightedness, and of the affections of the retina; with remarks on the use of Medicines as substitutes for Spectacles. By W. C. Wallace, oculist. Second Edition. —New York, 1839. Sm. 8vo, pp. 88.

53. Microscopic Illustrations of Living Objects, their Natural History, &c., with researches concerning the most eligible method of constructing microscopes, &c. By C. R. Goring, M.D. A new Edition, emended and enlarged. By A. Pritchard, M.R.I., &c.—London, 1840. 8vo, 248.

54. Elements of the Practice of Physic, presenting a view of the present state of special pathology and therapeutics. By D. Craigie, M.D., F.R.S.E., &c. Vol. Second. —Edin. 1840. 8vo, pp. 1256. 22s.

55. A Practical Treatise on the Functions and Diseases of the Unimpregnated Womb. Illustrated by Plates. With a chapter on Leucorrhœa. By C. Waller, M.D.—Lond. 1840. 8vo, pp. 200. 9s.

56. Organic Chemistry in its applications to Agriculture and Physiology. By Justus Liebig, M.D., &c. Edited from the manuscript of the author, by Lyon Playfair, PH. D.—London, 1840. 8vo, pp. 387. 12s.

FOREIGN.

1. De viribus et rationibus majorum dosium Calomellis. Auctore C. J. Æ. Hornemann, Medicus secundar. Nosocom. Frederic.—Hauniæ, 1839. 8vo, pp. 106.

2. Pharmacopœia Danica, regia auctoritate a collegio sanitatis regio Hafniensi edita.—Hafniæ, 1840. 8vo, pp. 316.

3. Dissertatio De Strabismo. Auctore N. G. Melchior.—Hauniæ, 1839. 8vo, pp. 75.

4. Memorie della Società medico-chirurgica di Bologna. Vol. II. Fasc. III. —Bologna, 1839.

5. Natuur-en geneeskundige Beschouwingen van moerassen en Moeraszickten, in verband met de vraag—Of de drookmaking van het Haarlemmermeer nadeelige gevolgen hebben zal voor de gezondheid der bewoners van nabijgelegene plaatsen en der arbeiders aan het meer. Door J. Van Geuns, M.D.—Amsterdam, 1839. 8vo, pp. 294.

6. Geneeskundige Bijdragen. Door A. A. Sebastian, M.D. Hoogleeraar in de Geneeskunde aan de Hoogeschool te Groningen.—Groningen, 1839. 8vo, pp. 218.

7. Bemerkungen über den Umgang mit Geisteskranken fur Diejenigen die mit ihnen in Berührung kommen. Von J. F. Lehmann.—Berlin, 1837. 8vo, pp 51.

8. De Haarlemmer-meer Quæstie. Door C. J. Van Cooth, Stads-doctor te Amsterdam.—Amsterdam, 1840. 8vo, pp. 148.

9. Recherches sur l'Introduction accidentelle de l'Air dans les Veines. Par I. L. Amussat.—Paris, 1839. 8vo, pp. 255.

10. Memoria sobre el plan de estudios, la organizacion y el personal de las Escuelas médicas estranjeras, con aplicaciones ā la nacional de San Carlos de Madrid. Par D. Melchior Sanches de Toca, M.D., &c. —Madrid, 1840. 4to, pp. 88.

11. Rapport sur l'Emploi des Eaux Minèrales de Vichy dans le traitement de la Goutte. Par M. Patissier.—Paris, 1840. 8vo, pp. 240.

12. Considérations sur la Structure de l'Encéphale et sur les rélations du crane avec cet organe. Par le Docteur Foville. Rapport de MM. Bouillaud, Bouvier, et Blandin.—Paris, 1840. 8vo, pp. 20.

13. Förhandlingar vid det af Skandinaviska Naturforskare och Läkare Hällna möte i Götheborg, är 1839.—Götheborg, 1840. 8vo, pp. 188.

14. Beschreibung einiger Fälle von anomaler Communication der Herzvorhöfe, &c. Von Dr. Alexander Ecker.—Freiburg, 1839. 8vo, pp. 86.

15. Pharmacopœia Castrensis Ruthenica. Auctore Jacobo Wylie, Equite Baronette, &c. &c. Editio Quarta.—Petropoli, 1840. Rev. 8vo, pp. 820.

16. Die häutige Bräune und die Gehirnenzündung, besonders jene der kinder. Von Dr. Ignaz R. Bischoff.

### INDEX TO VOL. X.

OF THE

### BRITISH AND FOREIGN MEDICAL REVIEW.

PAGE

Abortion, Mr. Streeter on . 548
Abscesses, urinary . . 574
Adams, Mr. on diseases of the joints 330
Air, necessity of in incubation . 229
Albuminuria, M. Solon on . 301
Alison, Dr. on vision . . 1
Amaurosis, Mr. Hocken on . 246
Amenorrhœa . . . 73
Ammon, Dr. Von on diseases of the eye 1
Anatomy of breast, Sir A. Cooper on 101
surgical, treatises on 347
Anchylosis of hipjoint, operation for 282
Andreæ, Dr. on ophthalmic medicine 1
Anæsthesia from lead . . 453
Angina pectoris, use of sulphur in 586
Antimonial vapours, poisoning by 580
Aorta, compression of, in flooding 274
Apoplexy, prevalence of, in Milan 425
Areola of the breast, anatomy of 114
Arsenic, sesquioxide of iron, antidote of . . . 287
Arteries, structure of . . 358
Articular cartilages, diseases of 338
Articulations, Mr. Blackburn on 330
Association, Irish Medical . 292
Provincial Medical . 293
Banks, Mr. on Medical Etiquette 215
Barlow, Dr. on albuminous urine 301
Belladonna, use of in eye-diseases 8
dysmenorrhœa 44
Bellows-sound, funic . . 274
Benevolent medical fund . 294
Black vomit in yellow fever . 278
Blackburn, Mr. on articulations 330
Blake, Dr. on delirium tremens . 551
Blood, menstrual, microscopic characters of . . . 257
Blood-vessels, relation of to muscles 354
on the contractility of 551
Boils, surgical anatomy of . 349
Bones, on the action of madder on 257
Books for review . . 300-600
Botany, Dr. Willshire on . 248
Brain, structure of cortical layer of 253
softening of . . 259
disease of, from disease of the heart . . 286
on the structure of . 296
affections of, in Bright's disease . . 318
compression of . 360
affections of from lead . 454
Breast, anatomy of . . 101
comparative anatomy of 128
fascia of the . . 115
Bright, Dr. on renal disease . 301

PAGE

Bright's disease, history of . 304
treatment of . 326
Bronchitis, on plastic . . 286
on acetate of lead in . 288
on diseases of the kidneys 317
Brodie, Sir B. on diseases of joints 330
Bubo, venereal, new mode of treating 418
Burne, Dr. on constipation . 40
Bushnan, Dr. on dysmenorrhœa. 593
Cæsarian section, case of . 578
Calculus, urethal, large . 264
Camel, on small-pox in . 281
Cartilages of joints, diseases of 330
Campbell, Dr. on extra-uterine gestation . . . 247
Cancer, Professor Müller on . 235
Carpus, dislocation of, forwards 51
Cartilages, articular, diseases of . 338
Cataract . . . 38
Ceely, Mr. on variolæ vaccinæ . 464
Chick, on the evolution of . 229
Chelius, M. on ophthalmology . 2
Children, facial hemiplegia of . 269
Cholera, on the progress of, in Britain 286
Christison, Dr. on disease of kidneys 301
Chorea, on cimicifuga in . 280
Churchill, Dr. on diseases of pregnancy 544
Cimicifuga, its use in cholera . 280
Cirrhosis of the liver, researches in 560
Clavicle, extirpation of . 265
excision of . . 568
Cod-liver oil, on iodine in . 558
Cod-oil, purification of . 584
Colic, lead, account of . 427
treatment of . 445
Combe, Dr. on infancy . 241-51
Consumption, on the treatment of 549
Corfe, Mr. on the kidney . 221
Conjunctiva, inflammation of . 31
Cornea, anatomy of . . 9
Crania Americana . . 474
Constipation, Dr. Burne on . 40
Cooper, Sir A. on the breast . 101
his discoveries . 102
Corns, application for the cure of 287
nature of . . 352
Cow, variolation of . . 469
Croup, treatment of . . 588
Cystocele, new operation for . 575
Daubeny, Dr. on education . 542
Deafness and dumbness from quinine 586
Deaths, sudden, statistics of in Milan 420
Delirium tremens, treatise on . 550
Dieffenbach, M. on strabismus 570
Dislocation of the carpus forwards 51
Diseases, new classification of . 57

PAGE

Doctors of the old and new school 300
Duparcque on ruptures of the uterus 486
Dwarf, case of labour in . 272
Dysmenorrhœa, use of belladonna in 44
Dr. Bushnan on 593
cure of by ointment of veratria 594
Ear, Dr. Williams on . 244
Eble, Dr. on Egyptian ophthalmy 3
Ectropium, operation for . 26
Education, medical, principles of 175
lectures on . 243
Dr. Daubeny on . 542
Egyptian ophthalmy, Eble on . 1
Elaterium, on the use of its principle 288
Empyema, observations on . 287
Encephalon, on the function of . 554
Etiquette, medical . . 215
Erysipelas of the eyelids . 25
Eye, numerous treatises on . 1
diseases of . . 1-20
examination of, by catoptrics 23-4
anatomy of . . 9
physiology of . . 13
on venereal diseases of . 393
Eyeball, diagnosis of enlargement of 585
Eyelids, erysipelas of . . 25
Fallopian tube, hernia of . 267
Feet, deformity of, cure of . 583
Femur, congenital luxations of . 576
Ferrario, Dr. on sudden deaths . 420
Fever, yellow, of Gibraltar . 494
morbid anatomy of . 496
yellow, attacks but once . 505
intermittent, on pain of back in 557
Fistula, vesico-vaginal, case of . 282
lachrymalis, treatment of 29
Flooding during labour . 84
compression of the aorta in 274
Fracture, mode of union of . 357
Fracture of the arm in labour . 577
Fuchs, Dr. on the effect of professions 373
Grave-yards, gatherings from . 212
Green, Mr. on vital dynamics . 545
Granular disease of the kidneys . 301
Graafian vesicles, changes in . 592
Gottenburg, scientific meeting at 549
Gibraltar, on the yellow fever of 494
Gestation, extra-uterine 247
influence of on the breast 123
Gendrin, M. his philosophical treatise 55
Gastrodynia . . 43
Galvanism in suspended animation 584
Hardwiger, Dr. on the ophthalmic clinic of Vienna . . 1
Harvey, on his doctrines . 238
Headach, sick . . 42
nervous . . 584
Heart, remarkable wound of . 49
on structure and functions of 238
disease of brain from disease of 286
diseases of, connected with Bright's disease . 316
congenital peculiarity of . 587

PAGE

Hearts, on the lymphatic of tortoises 256
Hemorrhage, M. Gendrin on . 59
causes of . 60
treatment of . 64
uterine . . 81
Hemorrhages, classification of . 59
Hemicrania and neuralgia, case of 262
Hemiplegia, facial, of infants . 269
Hemp, Indian, on the preparation of 225
Henle, Dr. on contractility of vessels 551
Hernia of the Fallopian tube . 267
inguinal, reduction of . ib.
new species of strangulated 565
radical cure of, by rest . 569
Heusinger, Dr. on the simia satyrus 251
Hipjoint, operation for anchylosis of 282
Hipjoint-disease, Dr. O'Beirne's treatment of . . 287
Humerus, congenital dislocation of 566
Hydriodate of potash, poisonous 588
Hydrocephalus, a peculiar case of 559
Hydrophobia, a new remedy in . 227
Hyoscyamus, use of, in ear-diseases 8
Idiocy and Imbecility . . 148
Idiocy, cure of from blow on head 277
Infancy, on the treatment of . 241
on moral management of 514
on the mortality in . 516
sources of disease in . 517
Infant, constitution of at birth . 519
clothing of . . 521
food of, at birth . 522
general management of . 525
Inflammation, treatment of . 532
Inoculation, variolous, abolition of 300
Inoculation of venereal diseases . 382
Insanity, medical jurisprudence of 129
Iodine in cod-liver oil . . 558
Ireland, Medical Association of . 292
Iris, on the physiology of . 10
anatomy of . . 12
Iritis, Dr. Von Ammon on . 2, 34
Mr. Lizars on . . 539
Iron, sesquioxide of, an antidote 287
lactate of, a new remedy . 565
sesquioxide in poisoning . 584
Itch, observations on . . 564
James, Mr. his retrospective address 463
Jaw, upper, removal of . 284
Joints, treatises on disease of . 330
Jones, Mr. W. on anatomy of the eye 9
Kay, Mr. on syphilitic cases . 381
Kidneys, Mr. Corfe's treatise on . 221
various treatises on diseases of 301
inflammation of . 302
abscess of . . 303
Bright's disease of . 304
Knee, M. Lederle on fungus of . 330
Labour of a dwarf, premature . 272
Lachrymal gland, diseases of . 28
Laura Bridgman, account of . 288
Lawrence, Mr. on venereal disease of the eye . . 381

52

PAGE
Lead, on the diseases from . 426
trades affected by . 431
Lee, Dr. on diseases of the uterus 249
the function of the ovaries 592
Leeches, danger of using old . 563
Leucorrhœa, Dr. Burne on . 44
Liver, on cirrhosis of . . 560
Library of Medicine by Dr. Tweedie 231
Lippich, Dr. his medical notes . 204
Lithotomy, M. Malgaigne on . 369
Liver, peculiar state of, in yellow fever 500
Lizars's, Mr. system of surgery . 537
London tables of mortality 289-589
Longevity in Russia . . 582
Louis, M. on the yellow fever . 494
Mackenzie, Dr. on diseases of the eye 3
syphilitic ophthalmia 381
Macula lutea of the eye, on the . 254
Madder, action of, on bones . 257
Malgaigne, M. on surgical anatomy 347
Malle, M. his clinical surgery . 49
Mammary gland, anatomy of . 116
Marc, M. jurisprudence of insanity 129
Mayo, Mr. on syphilis . 381
Medical study, observations on . 175
Medical systems, influence of on mortality . . 423
Medicine, philosophical treatise on 55
library of, by Dr. Tweedie 231
Membrana decidua, situation of . 587
Membranes, synovial, diseases of 331
Menstruation and its diseases . 67
theory of . 68
Dr. Lee on . 592
returning in old age 560
Mercury, use of, in diseases of the eye 8
Dr. Sigmond on . 245
its value in inflammation 532
Metro-hemorrhage . . 77
Microscopic illustrations of living objects . . . 550
Middlemore, Mr. on the eye . 3
Milan, topography of . . 421
on sudden deaths in . 420
Milk, on the secretion of . 124
chemistry of . . 127
Mind, unsoundness of . . 129
Monomania, homicidal . 139
Monstrosity, on a peculiar . 254-5
Morgan, Mr. on inflammation . 532
Mortality, London tables of 289-589
Morton, Dr. his crania Americana 474
Müller, M. on vision . 2
cancer . 235
Muscles, relation of nerves to . 352
blood-vessels to 354
rupture of . . 355
Muscular fibres, formation of . 260
Nævus, cure of, by vaccination . 26
Nephritis, M. Rayer on . 302
Nerves, their relation to muscles 352
Nervous system, illustrations of 508
Nervous fibres, on alterations of 553

PAGE
Neuralgia from lead . . 447
Neuralgia, use of strychnine in 588
Nevermann on rupture of the uterus 486
Nipple, anatomy of . . 111
Notes, medico-clinical, by Dr. Lippich 204
Odontography, Mr. Owen on . 208
Oils, essential, adulteration of . 582
Ophthalmia, variolous . 32
phlebitica . 33
sympathetic . ib.
intermittent . ib.
syphilitic, on . 381
strumous, on mercury in 584
Mr. Lizars on . 538
Ophthalmic medicine, treatises on 1
Ophthalmoscopy . . 28
O'Shaughnessy, Dr. on Indian hemp 225
Ovaries, Dr. Lee, on the function of 592
Owen, Mr. his odontography . 208
Padua, medical topography of . 205
Pagan, Dr. on insanity . 129
Pannus . . . 36
Parker, Mr. on treatment of syphilis 381
Penis, amputation of . 267
Perineum, surgical anatomy of 366
Peritonitis from injections into uterus 566
Paralysis from lead . . 449
Peru, on the diseases of 285-583
Phagedæna, syphilitic, treatment of 405
Phlebitis hepatica neonatorum . 580
Physiology, vegetable, report on 248
Pin-swallowing, curious case of 588
Placenta, on the position of . 285
Pneumonia, statistical researches on 262
on collapse in . 285
Pregnancy and childbed, disease of 544
Professions, effect of, on health . 373
Prolapsus ani, new operation for 568
Prostate gland, on the diseases of 528
Prostration, of operations during 587
Provincial Med. Association, Trans. of 461
Psoris, operation for . . 26
Puerperal fever and sloughing . 579
Pulse, Dr. Guy on the frequency of 288
Pupil, on the action of . . 10
artificial . . 29
Pustule, malignant, of the eyelids 26
Pyrosis, Dr. Burne on . . 43
Quinina, hydrocyanoferrate, a new febrifuge . . 584
Quinine, use of in diseases of the eye 8
causing deafness and dumbness 586
Ray, Dr. on the jurisprudence of insanity . . . 129
Rayer, M. on diseases of the kidneys 301
Rectum, supposed stricture of, fatal 45
Reflex motions, Dr. Nasse on . 257
Reform, medical . . 297
Renal disease, Dr. Bright on . 301
Respirator, account of . 591
Respiratory organs, affections of 583
Retina, structure of . . 12
action of . . 13

PAGE
Retrovaccination . . 468
Retroversion of the uterus . 286
Revaccination in the Prussian army 276
Rheumatism, a new remedy in 226-558
from lead . 447
use of sulphur in . 586
Ricord, M. on venereal diseases . 381
Rognetta, M. on ophthalmology . 2
Rötheln, account of at Leith . 285
Rudius, his pretended discoveries 238
Sanden, Dr. obituary of . 596
Scarlatina, its relation to the kidneys 319
Schumer, M. on diseases of cartilage 330
Schwann, M. on incubation . 229
Secale cornutum, on the action of 554
Shattuck's, Dr. translation of Louis 494
Shortsightedness, mode of curing 567
Sigmond, Dr. on mercury . 245
Simia satyrus, on the skull of . 251
Skin, Dr. Thomson on diseases of 250
wounds of, peculiarities of 351
on venereal diseases of . 381
Smallpox, affecting the eye . 32
in the camel . 281
in the bladder . 287
statistics of . 588
Softening of the brain, researches on 259
Solon, M. on albuminuria . 301
Sores, primary, syphilitic, treatment of . . . 398
Sormani, Dr. on sudden deaths . 420
Spinal cord, softening of . 52
Squinting, on the cure by operation 585-6
on the cure of . 288
Stafford, Mr. on the prostate gland 528
Staphyloma iridis . . 37
Statistics, medical, Danish . 583
Stereoscope, Mr. Wheatstone's . 18
Stomach, enlargement of . 583
St. Petersburg, mortality of . 582
Strabismus, on the operation for . 570-2
on the cure of . 573
on cure of, by operation 585-7
Streeter, Mr. on abortion . 548
Strychnine in neuralgia . 588
Sudden death, causes of . 424
Suicide, works on . . 129
Sulphur in spasmodic affections . 586
rheumatism . ib.
Surgery, clinical, of Strasburg . 49
Mr. Morgan's principles of 532
Mr. Lizars's system of . 537
Swan, Mr. on the nervous system 508
Symonds, Dr. his retrospective address 461
Synovial membranes, diseases of . 331
Syphilis, various treatises on . 381
primary, treatment of . 388
constitutional, treatment of 412
Tanquerel, Dr. on diseases from lead 426

PAGE
Tarsus, new mode of amputating . 271
Teeth, on the structure of . 209
Tetanus, a new remedy in . 227
Tendons, rupture of . . 355
Testicle, swelled, treatment of . 392
Thackeray prize . . 591
Thomson, Dr. on diseases of the skin 250
Throat, on a peculiar affection of 286
Todd, Dr. obituary of . 599
Tongue, on cramp of . . 557
Tortoises, on the lymphatic hearts of 256
Trismus in new-born infants, on . 275
Typhus, is it an exanthema? . 285
Ulcers, treatment of, in India . 284
Urine, incontinence of, new mode of treating . . . 287
Uterine hemorrhage . . 77
treatment of 79-94
Utero-placental hemorrhage . 81
Uterus, effects of constipation on 44
Dr. Lee on the diseases of 249
on retroversion of . 286
on ruptures of . . 486
Vaccination, a cure for nævus . 26
of the cow . 468
Vagina, on ruptures of . 486
Varicocele, new radical cure of . 270
Variola and varicella coincident 559
Variolation of the cow . 469
Variolæ vaccinæ, Mr. Ceely on 464
casual . ib.
Varix, on the treatment of . 577
Venereal diseases, treatises on . 381
Veins, on the obliteration of in varix 577
Velpeau, M. on diseases of the eyes 3
Velpeau, M. on surgical anatomy 347
Venereal sores, local treatment of 399
Veratria, ointment of . . 594
Vertebra, luxation of . . 567
Vertebral column, affections of . 363
Vidal, M. on diseases of the eye. 3
Vienna, ophthalmic clinic at . 1-5
Villards, M. De, on diseases of the eye 2
Vision, Alison, Wheatstone, &c. on 1
on single with two eyes . 16
Vital dynamics, Mr. Green on . 545
Walker, Mr. his work on churchyards 212
Wallace, on the venereal disease . 381
Wheatstone, Mr. on vision . 1
Williams, Dr. on the ear . 244
Willshire, Dr. on botany . 248
Wilson's, Mr. E. anatomical vade mecum 547
Woollen manufactures, healthy . 587
Wound, gunshot, relative size of 53
on subcutaneous . 265
cutaneous, nature of . 351
Xerophthalmia . . 36
Yellow fever, on black vomit in . 278
on quinine in . 278

PRINTED BY C. ADLARD, BARTHOLOMEW CLOSE.

